# Macrophage production and activation are dependent on TRIM33

**DOI:** 10.18632/oncotarget.13872

**Published:** 2016-12-10

**Authors:** Anne-Sophie Gallouet, Federica Ferri, Vanessa Petit, Aude Parcelier, Daniel Lewandowski, Nathalie Gault, Vilma Barroca, Stéphanie Le Gras, Eric Soler, Frank Grosveld, Irwin Davidson, Paul-Henri Romeo

**Affiliations:** ^1^ CEA/DRF/iRCM/LRTS, 92265 Fontenay-aux-Roses cedex, France; ^2^ Inserm U967, 92265 Fontenay-aux-Roses cedex, France; ^3^ Université Paris-Diderot, Paris 7, France; ^4^ Université Paris-Sud, Paris 11, France; ^5^ Equipe labellisée Ligue contre le Cancer, France; ^6^ Department of Functional Genomics and Cancer, Institut de Génétique et de Biologie Moléculaire et Cellulaire, CNRS/INSERM/ULP, BP 163, 67404 Illkirch Cedex, C. U. Strasbourg, France; ^7^ Department of Cell Biology, Erasmus Medical Center, DR Molenwaterplein 50, 3015GE, Rotterdam, The Netherlands

**Keywords:** TRIM33, PU.1, macrophage, inflammation, myeloid differentiation

## Abstract

The tripartite motif (TRIM) family of proteins plays important roles in innate immunity and antimicrobial infection. None of these proteins has been shown to directly regulate transcription of genes in monocyte/macrophage except TRIM33 that we have recently shown to be a macrophage specific transcriptional inhibitor of *Ifnb1*. Using ChIP-seq analyses, we now report that TRIM33 is bound to two fold more genes in immature than in mature myeloid cell lines. When located near the same genes, TRIM33 is bound to different sequences in the two cell lines suggesting a role of TRIM33 in both immature and mature myeloid cells. Accordingly, expression of TRIM33 in immature myeloid cells is necessary for efficient production of small peritoneal macrophages, monocytes and bone marrow derived macrophage (BMDM) and TRIM33 targets a subset of genes involved in the inflammatory response only in mature myeloid cells. Functionally, this targeting is associated with impaired repression of pathways regulating the late phases of lipopolysaccharide (LPS) activation of BMDM and a high sensitivity to LPS *in vivo* when the *trim33* gene is inactivated in mature myeloid cells. These findings pinpoint TRIM33 as an important transcriptional actor of monocyte/macrophage mediated inflammation.

## INTRODUCTION

Inflammation requires coordinated cellular interactions between a variety of different cell types belonging to innate and adaptive immune systems and parenchymal cells surrounding the site of inflammation [1]. Macrophages are mobile hematopoietic cells that act as immune sentinels [2]. They reside in most organs and can deeply migrate within a tissue to the focus of inflammation [3]. In response to extracellular signals that activate their respective receptors, macrophages transcriptionally activate a complex network of genes and become one of the principal cellular effectors of acute and/or chronic inflammation [4]. The initial activation state of macrophages results in synthesis of pro-inflammatory mediators with antimicrobial and/or antiviral effects, but persistent expression of these mediators can cause extensive tissue damage [5, 6]. Thus, macrophage activation is transitory and can be resolved into a state such as bacterial tolerance, where pro-inflammatory mediator genes are silenced while antimicrobial effector genes are primed [7].

Tripartite motif (TRIM) family proteins share an N-terminal tripartite motif that consists of a RING domain, one or two B-boxes and a coiled-coil domain and most TRIM family members have an E3 ubiquitin ligase activity. Numerous members of the TRIM family have been implicated in various innate signaling pathways both in the cytoplasm and in the nucleus. TRIM33 (TIF1γ, Ectodermin) belongs to a sub-family of chromatin binding TRIM proteins [8] that also includes TRIM24 (TIF1α), TRIM28 (TIF1β, KAP1) and TRIM66 (TIF1δ). The importance of TRIM33 in hematopoiesis was revealed by a genetic study of bloodless zebrafish mutants called *moonshine* that display cell-autonomous defects in primitive and definitive adult hematopoiesis [9]. In adult mice, targeted deletion of *Trim33* in hematopoietic stem cells (HSC) affects the HSC compartment and promotes the expansion of Granulocyte-Monocyte Progenitors (GMP) at the expense of Common Myeloid Progenitors (CMP) and Megakaryocyte-Erythrocyte Progenitors (MEP) [10–12]. At the molecular level, interactions between TRIM33 and GATA1, TAL1 or PU.1 regulate the function of these hematopoietic regulators [12, 13].

The PU.1/TRIM33 interaction together with the role of PU.1 in the chromatin changes that occur during macrophage production and in macrophages before and during the initiation of the inflammatory response [14] and the hematopoietic phenotype of mice deficient for TRIM33 in hematopoietic cells suggest that TRIM33 may regulate myeloid fate and have a role in macrophage. Recently, three studies have indicated such a role of TRIM33. TRIM33 was shown (i) to interact and ubiquitinate DHX33 and be essential for the DHX33-NLRP3 inflammasome complex [15], (ii) to bind an *Ifnb1* regulatory region that acted as a repressor of the *Ifnb1* gene at the end of bone marrow derived macrophage (BMDM) activation by LPS [16] and (iii) to be involved in late stages of granulomonopoiesis [17]. Here, we characterize the role of TRIM33 in macrophage production and activation using chromatin immunoprecipitation (ChIP) coupled to deep sequencing (ChIP-seq) analyses and two mouse models of *Trim33* inactivation.

## RESULTS

### Differential TRIM33 chromatin binding in immature *versus* mature myeloid cell lines

**Figure 1:**
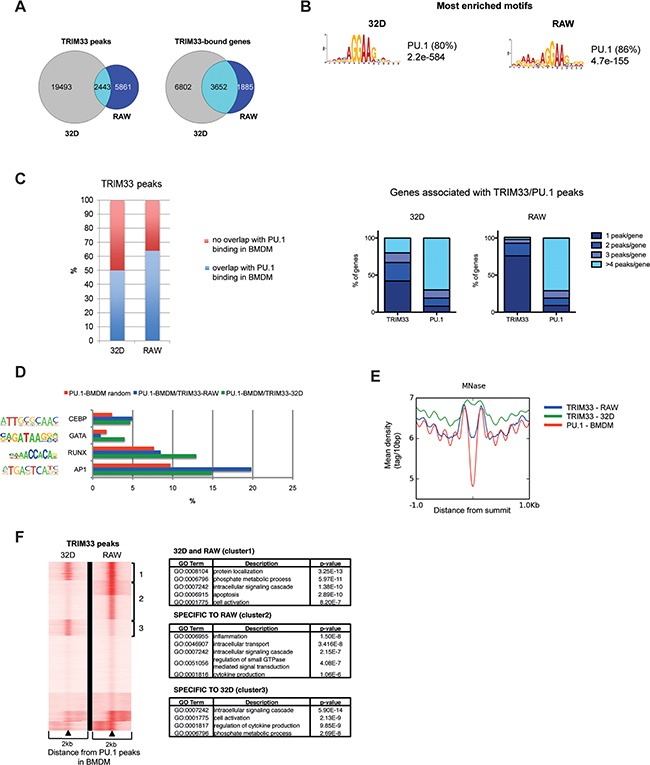
Dynamics of TRIM33 binding in myeloid cell lines **A**. Venn diagrams of TRIM33 peaks (left) and associated genes (right) in 32D and RAW cells. The number of cell-specific or co-localized peaks and shared genes are shown for each cell line. **B**. Most enriched sequence motifs in TRIM33 peaks in 32D and RAW cells. E-values on the significance of the motifs are given. The percentages of non-overlapping sites (p< 0.0001) are given in parentheses. **C**. (Left) Overlap between TRIM33 peaks in 32D and RAW cells and PU.1 peaks in BMDM (data sets from [18]). (Right) Distribution of genes according to their numbers of associated TRIM33 and PU.1 peaks in 32D and RAW cells. **D**. Occurrence of the indicated transcription factor binding sites in the different TRIM33/PU.1 peak subsets in 32D (green) and RAW (blue) cells compared to their frequency in random PU.1-bound regions in BMDM (red). Transcription factor matrices were extracted from Homer database and analyzed with RSAT Matrix-scan, using a threshold p-value<1e-4. **E**. MNase profiles in BMDM (data sets from [19]) around the summit of TRIM33 peaks in 32D (blue) or RAW (green) cells and around the summit of PU.1 peaks in BMDM (red). **F**. (Left) PU.1 binding sites in macrophages were clustered according to their TRIM33 ChIP-seq profiles in 32D and RAW cells. (Right) Gene Ontology (GO) functional annotation analysis of the three clusters of TRIM33/PU.1 target genes.

To get insight into the role of TRIM33 in myeloid cells, we first studied, by ChIP-seq, TRIM33 binding to chromatin in murine immature myeloid 32D and monocyte/macrophage RAW 264.7 (RAW) cell lines. This analysis identified 21936 and 8304 TRIM33 peaks associated with 10454 and 5537 genes in 32D and RAW cells respectively (Figure 1A). TRIM33 peaks were enriched at promoter/transcriptional start sites (TSS) (Supplementary Figure S1A, left panels), with a higher TRIM33 occupancy of the TSS in RAW compared to 32D cells (Supplementary Figure S1A, right panels). The proportion of overlapping TRIM33 peaks between the two cell types was relatively small (2443 peaks), representing 11% of total peaks in 32D cells and 29% of total peaks in RAW cells. However, analysis of genes associated with the nearest TRIM33 peak showed that the number of common TRIM33-bound genes was higher than expected regarding the number of overlapping peaks. It corresponded to 3652 genes, i.e. 35% and 66% of total TRIM33-bound genes in 32D and RAW cells (Figure 1A, right Venn diagram, Supplementary Figure S1B). Altogether, these results show a higher number of TRIM33 binding sites in immature myeloid cells and different binding sites of TRIM33 on same genes in immature and mature myeloid cells.

To characterize the TRIM33 peaks in immature and mature myeloid cells, we performed *de novo* motifs analysis. The PU.1 binding motif was the most enriched motif found at TRIM33 peaks in 32D (80%) and RAW (86%) cells (Figure 1B). Comparison with published PU.1 ChIP-seq data from BMDM [18] showed that 50% and 65% of TRIM33 peaks in 32D and RAW cells, respectively, overlapped with PU.1 binding sites in BMDM (Figure 1C, left panel). Finally, in 32D cells, 42% of genes that shared TRIM33 and PU.1 binding sites had a single TRIM33 peak whereas 92% of these genes had more than one PU.1 peak in BMDM (Figure 1C, right panels). In RAW cells 76% of genes that shared TRIM33 and PU.1 binding sites had a single TRIM33 peaks whereas 91% of these genes exhibited multiple PU.1 peaks (Figure 1C, right panels). Altogether, these results indicate that TRIM33 might be recruited on specific subsets of PU.1 binding sites in 32D and RAW cells.

To identify sequence determinants of these subsets of TRIM33/PU.1 binding sites, we investigated the occurrence of several myeloid-determining transcription factors motifs within 100bp of these binding sites and compared with their occurrence in randomly selected PU.1 peaks. In 32D cells, this analysis showed that TRIM33/PU.1 binding sites were enriched for GATA, RUNX, CEBP motifs and to a lesser extend AP1 motifs whereas, in RAW cells, TRIM33/PU.1 sites were enriched in motifs for CEBP and AP1 motifs (Figure 1D and Supplementary Figure S1C for control motifs). TRIM33 binding at PU.1 binding sites might therefore require binding of different lineage-determining transcription factors in immature and mature myeloid cells.

As PU.1 can maintain nucleosome depletion at macrophage-specific enhancers [19], we analyzed association of TRIM33 peaks found in 32D and RAW cells with nucleosome occupancy in BMDM. We found that TRIM33 peaks correlated with nucleosome-free regions associated with PU.1 binding in BMDM only in RAW cells (Figure 1E), a property highly increased when TRIM33/PU.1/AP1 binding sites were analyzed (Supplementary Figure S1D). This result indicated that TRIM33 might be recruited at nucleosome free macrophage-specific regions enriched in PU.1/AP1 binding sites in mature myeloid cells.

Integrative analysis of TRIM33 ChIP-seq data in 32D and RAW cells and PU.1-occupied sites in BMDM identified at least three major clusters (Figure 1F, left panel). Interestingly, one of these clusters, corresponding to TRIM33/PU.1 binding sites specific to RAW cells, was highly enriched in genes expressed in monocytes and macrophages and involved in inflammatory response (Figure 1F, right panels and Supplementary Figure S1E) suggesting a specific role of TRIM33 in macrophages.

### TRIM33 deficiency at early stages of hematopoiesis is associated with impaired monocyte/macrophage production

**Figure 2:**
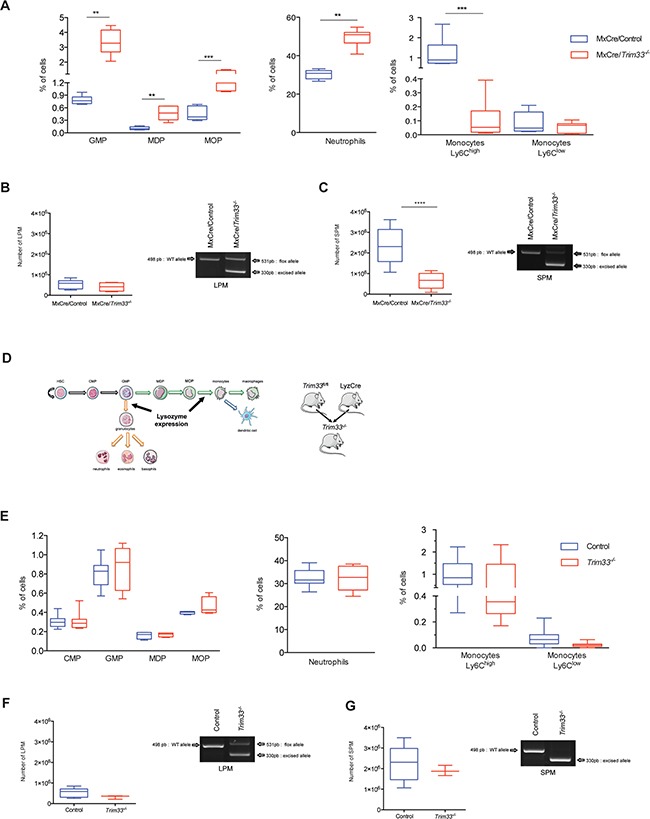
Role of TRIM33 during myeloid differentiation **A**. Box plots of indicated bone marrow (BM) hematopoietic populations of MxCre/Control and MxCre/*Trim33^−/−^* mice. GMP (Granulocyte-Macrophage Progenitor) (Lin^−^, Sca1^−^, c-Kit^+^, CD16/32^+^, CD34^+^), MDP (Monocyte/Macrophages and Dendritic cell Precursor) (Lin^−^, CD115^+^, CD117^+^, CD135^−^), MOP (common MOnocyte Precursor) (Lin^−^, CD115^+^, CD117^+^, CD135^+^), neutrophils (CD11b^+^, (F4-80,I-A(b), CD11c)^med^, Ly6G^high^), and monocytes (SSC^low^, B220^−^, (F4-80,I-A(b), CD11c^low^), CD115^+^, Ly6C^high/low^). n=9-12 mice, ***p<0.0006, **p<0.001, *p<0.02 (Mann-Whitney test). **B**. (Left) Total number of Large Peritoneal Macrophages (LPM), (B220^−^, CD11c^−^, CD11b^+^, F4-80^high^) in MxCre/Control and MxCre/*Trim33^−/−^* mice. n=4-9 mice, ***p<0.0002 (Mann-Whitney test). (Right) Genomic PCR from MxCre/Control and MxCre/*Trim33^−/−^* LPM for *Trim33* floxed and *Trim33* excised allele. Only relevant bands are shown. **C**. (Left) Total number of Small Peritoneal Macrophages (SPM) (B220^−^, CD11c^−^, CD11b^+^, F4-80^+^) with thioglycollate induced peritonitis in MxCre/Control and MxCre/*Trim33^−/−^* mice. n=4-9 mice, ***p<0.0002 (Mann-Whitney test). (Right) Genomic PCR from MxCre/Control and MxCre/*Trim33^−/−^* SPM for *Trim33* floxed and *Trim33* excised allele. Only relevant bands are shown. **D**. Schema of myeloid differentiation, Lysozyme expression and generation of *Trim33^−/−^* mice. HSC (Hematopoietic Stem Cell) and CMP (Common Myeloid Progenitor) **E**. Box plots of indicated hematopoietic populations from BM of  Control and *Trim33*^−/−^ mice. n=8-15 mice. **F**. (Left) Total number of large peritoneal macrophages (LPM) in Control and *Trim33^−/−^* mice. n=3-9 mice. (Right) Genomic PCR from Control and *Trim33^−/−^* LPM for *Trim33* floxed and *Trim33* excised allele. Only relevant bands are shown. **G**. (Left) Total number of small peritoneal macrophages (SPM) with thioglycollate induced peritonitis in Control and *Trim33^−/−^* mice. n=3-9 mice. (Right) Genomic PCR from Control and *Trim33^−/−^* SPM for *Trim33* floxed and *Trim33* excised allele. Only relevant band are shown.

To characterize the role of TRIM33 in immature and mature myeloid cells, we first used MxCre/*Trim33^−/−^* mice where deletion of *Trim33* is induced, in all hematopoietic cells, by PIPC treatment of adult mice. One month after PIPC treatment, in their bone marrow (BM), MxCre/*Trim33^−/−^* mice had increased percentages of Granulocyte-Macrophage Progenitor (GMP) [12], but also of Monocyte/Macrophages and Dendritic cells Precursor (MDP) and of common MOnocyte Precursor (MOP) compared to MxCre/*Trim33^+/fl^* mice treated with PIPC (named MxCre/Control hereafter) (Figure 2A, left panel). As for mature myeloid cells, these mutant mice had an increased percentage of neutrophils (Figure 2A, middle panel), a ten-fold decreased percentage of Ly6C^high^ monocytes and similar percentage of Ly6C^low^ monocytes (Figure 2A, right panel), both populations harboring complete deletion of *Trim33* (Supplementary Figure S2A). In accordance with the BM cellular composition, MxCre/*Trim33^−/−^* mice displayed, in peripheral blood cells, a six-fold decreased percentage of inflammatory monocytes Ly6C^high^, a two-fold increased percentage of neutrophils and a two-fold decreased percentage of B lymphocytes when compared to MxCre/Control mice (Supplementary Figure S2B).

To analyze the effect of TRIM33 deficiency on resident and inflammatory macrophages, we studied the peritoneal macrophages population that contains large peritoneal macrophages (LPM), derived from embryonic precursors and/or monocytes, and small peritoneal macrophages (SPM), derived from monocytes during inflammation [20]. MxCre/Control and MxCre/*Trim33^−/−^* mice had a similar number of LPM in the peritoneal cavity (Figure 2B, left panel) and, in accordance with their origin, these LPM only harbored a partial deletion of *Trim33* (Figure 2B, right panel). After thioglycollate-induced peritonitis, the number of SPM was three-fold reduced in MxCre/*Trim33^−/−^* mice compared to MxCre/Control mice (Figure 2C, left panel) and these SPM had a complete deletion of *Trim33* (Figure 2C, right panel). Altogether, these results suggest that TRIM33 deficiency in hematopoietic cells was associated with impaired production of monocytes/macrophages.

When MxCre/Control and MxCre/*Trim33^−/−^* BM were grown in the presence of CSF-1 to generate bone marrow derived macrophages (BMDM), a decreased production of BMDM from MxCre/*Trim33^−/−^* BM was found (Supplementary Figure S2C, upper panel). This decrease correlated with decreased number of monocytes Ly6C^high^ found in MxCre/*Trim33^−/−^* BM (Figure 2A, right panel). Although MxCre/*Trim33^−/−^* macrophages were F4-80^+^/CD64^+^, their morphology was different from MxCre/Control macrophages (Supplementary Figure S2C, lower panel). To study the myeloid differentiation of MxCre/*Trim33^−/−^* BM progenitors, we investigated their differentiation in the presence of GM-CSF or G-CSF. In the presence of GM-CSF, less clusters of dendritic cells and less F4-80^+^/CD64^+^ macrophages were obtained with MxCre/*Trim33^−/−^* BM (Supplementary Figure S2D) but MxCre/*Trim33^−/−^* and MxCre/Control derived dendritic cells had similar morphology (not shown). When MxCre/*Trim33^−/−^* BM was grown in the presence of G-CSF, very few F4-80^+^/CD64^+^ macrophages could be obtained with MxCre/*Trim33^−/−^* BM (Supplementary Figure S2E, upper panel). Altogether, these results indicate that the decreased number of macrophages found is correlated with the decreased number of monocytes found in MxCre/*Trim33^−/−^* BM but also suggest that TRIM33 deficiency in hematopoietic cells diminished their capacity to produce macrophages.

To study the role of TRIM33 specifically in mature myeloid cells, *Trim33^fl/fl^* mice were crossed with mice expressing the Cre recombinase gene from the endogenous *Lysozyme2* locus (LyzCre). In this mouse model (hereafter referred to as *Trim33^−/−^* mice), *Trim33* was targeted only in mature myeloid cells (Figure 2D) [21]. *Trim33^−/−^* mice did not display developmental abnormalities and were healthy. No difference in percentage of myeloid progenitors/precursors and mature myeloid cells could be detected in *Trim33^−/−^* and LyzCre Control BM (Figure 2E). LyzCre Control (named Control hereafter) and *Trim33^−/−^* mice had the same number of LPM in the peritoneal cavity (Figure 2F, left panel), the majority of which harboring deletion of *Trim33* (Figure 2F, right panel). After thioglycollate-induced peritonitis, the number of SPM in Control and *Trim33^−/−^* mice was similar (Figure 2G, left panels) and these SPM were all deleted for *Trim33* (Figure 2G, right panel). Finally, BMDM obtained from Control and *Trim33^−/−^* BM were similar both in number (not shown) and morphology (Supplementary Figure S2F).

Altogether, these results show that TRIM33 expression at early but not late stages of myeloid differentiation is necessary for efficient production of monocytes/macrophages.

### Characterization of gene expression during LPS activation of *Trim33^−/−^* BMDM

**Figure 3:**
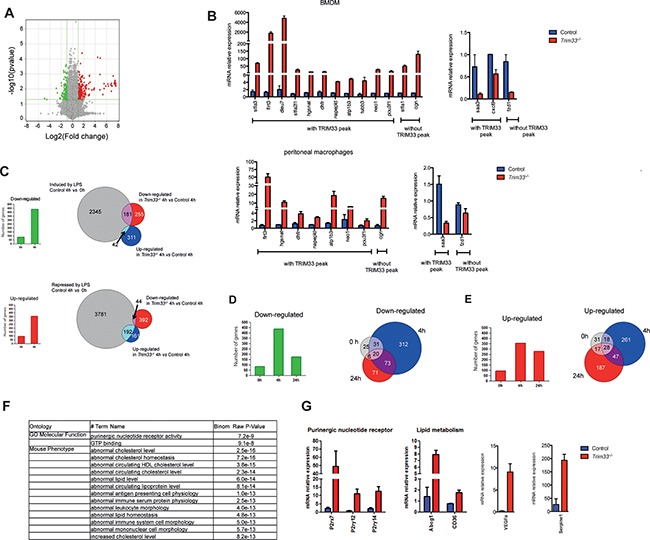
TRIM33 regulates gene expression of a subset of genes in BMDM **A**. Gene expression changes in *Trim33^−/−^ versus* Control BMDM (green: down-regulated genes; red: up regulated genes; FC 2; p<0.05). **B**. Relative mRNA levels in BMDM (top) and thioglycollate-elicited peritoneal macrophages (PM) (bottom) of genes with the highest FC (FC>4, p<0.05) were measured by quantitative RT-PCR. Data are the average fold changes relative to Control BMDM or PM ± SEM. Genes harboring TRIM33 peaks in RAW cells are indicated. **C**. Number of down- and up-regulated genes (FC 2; p<0.05) in *Trim33^−/−^* BMDM before (0h) and 4h after LPS activation (left histograms). Overlap between down-regulated or up-regulated genes in *Trim*33^−/−^ BMDM and LPS-induced (upper panel) or -repressed (lower panel) genes 4 hours after LPS activation. **D**. (Left) Number of down-regulated genes (FC 2; p<0.05) in *Trim33^−/−^* BMDM at indicated time points after LPS activation and (right) overlapping of down-regulated genes before (0h), 4h and 24h after LPS activation of BMDM. **E**. (Left) Number of up-regulated genes (FC 2; p<0.05) in *Trim33^−/−^* BMDM at indicated time points after LPS activation and (right) overlapping of up-regulated genes before (0h), 4h and 24h after LPS activation of BMDM. **F**. GREAT functional annotation analysis of the genes harboring TRIM33 peaks in RAW cells and up-regulated (FC 2; p<0.05) in *Trim33^−/−^* BMDM 24h after LPS activation. **G**. Relative mRNA levels in BMDM of indicated genes measured by quantitative RT-PCR after 24h of LPS activation. Mean ± SEM, n=3.

ChIP-seq analysis in RAW and 32D cells identified genes containing PU1/TRIM33 binding sites and involved in the inflammatory response only in RAW cells (Figure 1F and Supplementary Figure S1E). These results indicated that, in addition to its role in the production of monocytes/macrophages, TRIM33 might also shape the transcriptional activity of macrophages before and/or after activation. We therefore used *Trim33^−/−^* mice to globally characterize the role of TRIM33 in BMDM before and after LPS activation, using cDNA microarrays. In the absence of activation, 82 transcripts were down-regulated and 93 up-regulated (Fold change FC 2, p<0.05) in *Trim33^−/−^* compared to Control BMDM (Figure 3A and Supplementary Table S1). Most of the genes with the highest FC (FC 4, p<0.05) were up-regulated in *Trim33^−/−^* BMDM and more than 75% of these genes had at least one associated TRIM33 peak in RAW cells (Supplementary Table S2). Indeed, ChIP-qPCR analysis in immortalized *Trim33^−/−^* macrophages rescued with full-length TRIM33 protein showed TRIM33 binding to these genes in macrophages (Supplementary Figure S3A). The changes in expression of genes with FC>4 were confirmed by RT-qPCR in BMDM (Figure 3B, upper panels) and in thioglycollate-elicited peritoneal macrophages (Figure B, lower panels). Altogether, these results indicate that TRIM33 mainly acts as a transcriptional repressor in macrophages *in vitro* and *in vivo*.

As cell surface expression of TLR4 was similar in Control and *Trim33^−/−^* BMDM (Supplementary Figure S3B), we studied the consequences of TRIM33 deficiency during LPS activation of BMDM. Microarray analysis (FC 2, p<0.05) showed that, 4h after LPS activation, the number of deregulated genes in LPS-activated *Trim33^−/−^* BMDM represented a small percentage (7%) of the genes induced or repressed by LPS treatment in Control BMDM (Figure 3C, right panels). This result indicated that early TLR4 triggered signaling pathways were not grossly modified by TRIM33 deficiency. Accordingly, early kinetics of the level of mRNAs coding for several inflammatory cytokines or known regulators of macrophage functions were similar in Control and *Trim33^−/−^* BMDM activated with LPS (Supplementary Figure S3C).

Four hours after LPS activation of *Trim33^−/−^* BMDM, the total number of down-regulated genes increased 5-fold and the total number of up-regulated genes increased 3-fold when compared to non-activated BMDM (Figure 3C, left panels). Interestingly, down-regulated genes were overrepresented among those induced by LPS in Control BMDM (Figure 3C, upper right panel), whereas up-regulated genes were overrepresented among those repressed after LPS activation (Figure 3C, lower right panel). These results indicated that TRIM33 deficiency in BMDM was associated with an impaired transcriptional response of a subset of genes in the early response to LPS. To characterize this subset, a Gene Ontology analysis was performed and showed a specific enrichment of genes involved in wound healing, such as Nos2 (down-regulated genes) or in organelle fission, such as Fis1 (up-regulated genes) (Supplementary Figure S3D).

At the end of LPS activation of BMDM, i.e. 24 hours after this activation, 170 transcripts were down-regulated and 279 up-regulated in *Trim33^−/−^* BMDM. Compared to 4 hours, the number of down-regulated genes in *Trim33^−/−^* BMDM (FC 2, p<0.05) at 24 hours decreased (Figure 3D, left panel) and 60% of these genes were already down-regulated before or 4 hours after LPS activation (Figure 3D, right panel and Table S3). In contrast, the number of up-regulated genes in *Trim33^−/−^* BMDM was similar after 4 and 24 hours of LPS activation (Figure 3E, left panel), but only 30% of the genes up-regulated at 24 hours were up-regulated before or 4 hours after LPS activation (Figure 3E, right panel and Table S3). This indicated that TRIM33 might be required for repression of a distinct subset of genes at the end of LPS stimulation of BMDM.

Microarray analyses and TRIM33 ChIP-seq data in RAW cells, highly correlated with TRIM33 binding in BMDM 24 hours after LPS activation (Supplementary Figure S3E), showed that 30% (83 out of 279) of genes up-regulated 24 hours after LPS activation of *Trim33^−/−^* BMDM were associated with at least one TRIM33 peak (Table S3) and 86% of these TRIM33-target genes shared a PU.1/TRIM33 binding site (Table S3). GREAT analysis of these TRIM33-target genes showed a specific enrichment of genes involved in purinergic nucleotide receptor activity and in lipid metabolism (Figure 3F), including important regulators at the end of inflammatory response of BMDM, such as P2rx7, P2ry12, P2ry14, Abcg1, Cd36, Serpine1, VEGFa, Esr1 and OX40 ligand (Tnfsf4) (Figure 3G and Supplementary Figure S3F).

Altogether, these results indicate impaired repression of pathways regulating the late phases of lipopolysaccharide (LPS) activation of BMDM in TRIM33 deficient BMDM.

### Impaired response of *Trim33^−/−^* mice to an endotoxin challenge

**Figure 4:**
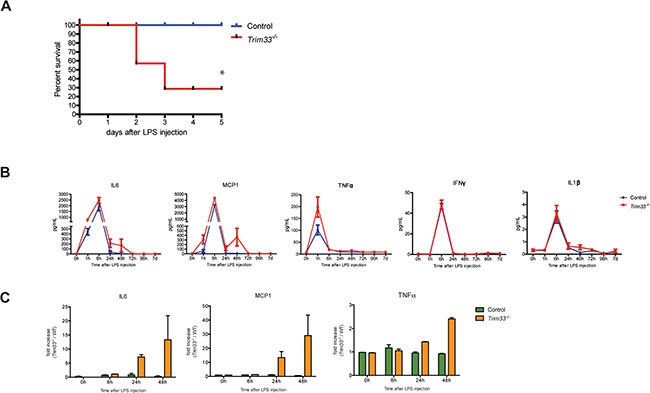
TRIM33 deficiency is associated with high sensitivity to endotoxin challenge **A**. Survival curves of Control and *Trim33*^−/−^ mice after intra-peritoneal injection of LPS (0.25mg/20g). (n=8-9 mice for each group). * p<0.02 (Gehan-Breslow-Wilcoxon test). **B**. Kinetics of serum concentrations of the indicated cytokines after intra-peritoneal injection of LPS. Mean ± SEM, n=5 mice. **C**. Fold increase of IL6, MCP1 and TNFα in serum of dying *Trim33^−/−^* mice *versus* Control mice (serum collected just before the death of *Trim33^−/−^* mice) (n=3 *vs* 5).

The effects of TRIM33 deficiency on gene expression in BMDM and PM suggested that the *in vivo* response of *Trim33*^−/−^ mice to LPS might be altered. We therefore treated Control and *Trim33^−/−^* mice with a sub-lethal dose of LPS. All Control animals survived to this endotoxin challenge, whereas only 28% of *Trim33^−/−^* mice survived (Figure 4A). This decreased survival was not associated with any difference in the plasma levels of IL6, MCP-1, TNFα, IFNγ and IL1β (Figure 4B) in the early phase of cytokine production. However, mortality was associated with increased plasma levels of IL6, MCP-1 and TNFα during the late phase of the endotoxin challenge in *Trim33^−/−^* mice (Figure 4C) suggesting impaired functions of LPS activated *Trim33^−/−^* myeloid cells at the end of this endotoxin challenge.

## DISCUSSION

TRIM33 has essential roles in mouse hematopoietic stem cells and multiple blood lineages and is involved in macrophage function and inflammation. Indeed, TRIM33 has been shown to regulate NLRP3 inflammasome activation in response to bacterial or viral infection [15] through a direct interaction with DHX33 in the cytoplasm and to regulate *Ifnb1* transcription at the late stages of BMDM activation [16]. Here, we extend this role by showing that TRIM33 regulates the production of macrophages and is also involved in the innate immune response by the regulation of a subset of genes in BMDM and primary macrophages activated with LPS.

ChIP-seq analysis of TRIM33 in murine immature and mature myeloid cell lines showed that TRIM33 recruitment was enriched over the transcription start site, a property also found in testis [22]. TRIM33 is not thought to bind DNA directly and our study revealed a preferential association of TRIM33 with PU.1 binding sites in 32D and RAW cell lines. Recently, ChIP-seq analysis of TRIM33 in murine B cell leukemia (B-ALL) has shown that TRIM33 is concentrated, in the B-ALL genome, at a small number of sites characterized by a high density of sequence motifs recognized by PU.1 [23]. Our analysis in 32D and RAW cell lines revealed that TRIM33 was mostly recruited on a large subset of PU.1 binding sites flanked by motifs for different myeloid-determining factors, including GATA, RUNX, CEBP in 32D cells and CEBP and AP1 in RAW cells. This discrepancy might be related to a different recruitment of TRIM33 in myeloid and B-lymphoid cells, discrepancy related to the different transcription factors that increased TRIM33 recruitment on specific PU.1 sites.

The two strongest sites of TRIM33 enrichment in B-ALL were located 117bp upstream of *Bcl2l11/Bim* gene (in an intron of *Acoxl* gene), and at a site 35 kb upstream of *Atp1b3*. We also found TRIM33 recruitment to these sequences in 32D and RAW cell lines, with additional TRIM33 peaks at the Bcl2l11/Bim locus in myeloid compared to lymphoid cells (Supplementary Figure S4A). In B-ALL cells, *Atp1b3* and *Bcl2l11/Bim*, but not *Acoxl*, genes expression is highly increased upon TRIM33 knock-down. TRIM33 deficiency in BMDM and peritoneal macrophages was also associated with an increased expression of *Atp1b3 and Bcl2l11/Bim* (Table S3). Interestingly, *Acoxl*, although only weakly expressed in Control BMDM, was strongly up-regulated in activated *Trim33^−/−^* BMDM. Altogether, these results suggest a repressive effect of TRIM33 on the expression of *Atp1b3* and *Bcl2l11/Bim* genes both in myeloid and lymphoid lineages. However, as illustrated by the *Acoxl* gene regulation, the mechanisms of repression might be different and related to different chromatin structure in the two lineages.

TRIM33 deficiency in immature myeloid cells impaired the production of monocytes/macrophages both *in vitro* and *in vivo*. This result is in accordance with a recent study that showed that *Trim33* knock-out in hematopoietic cells results in abnormal monocyte and macrophage maturations associated with decreased expression of the CSF-1 receptor [17]. We did not find CSF1-R decreased expression in MxCre/*Trim33^−/−^* mice (data not shown) although TRIM33 peaks were present in the *Csf1-R* gene with one peak within the *Csf1-R* intronic enhancer [24] (Supplementary Figure S4B). This discrepancy might be explained by the two conditional hematopoietic-specific *Trim33* knock-out used. We used MxCre/*Trim33^−/−^* mice where *Trim33* knock-out is induced by PIPC treatment of adult mice and whereas Chrétien et al. used cFES-Cre where *Trim33* knock-out is constitutive during adult hematopoiesis and during development of the hematopoietic tissue. This differential Cre expression results in leukemia in the cFES-Cre mediated *Trim33* knock-out whereas MxCre/*Trim33^−/−^* mice never developed myelomonocytic leukemia. The decreased expression of the CSF-1 receptor might thus be related to this leukemic transformation.

TRIM33 deficiency in mature myeloid cells did not severely impair macrophage production and specific property of macrophages such as phagocytosis capability (data not shown). In addition, TRIM33 deficiency did not alter the early TLR4 triggered signaling pathways as shown by similar early kinetics of the level of mRNAs coding for several inflammatory cytokines or known regulators of macrophage functions in Control and *Trim33^−/−^* BMDM activated with LPS. Recently, TRIM33 has been shown to be essential for the cytosolic dsRNA-induced NLRP3 inflammasome activation by targeting DHX3 [15]. As NLRP3 is activated by various stimuli including LPS plus ATP treatment, but not by LPS treatment alone, these results indicated that TRIM33 might have different functions depending on the type of activation of BMDM.

Contrary to the early phases of LPS activation of BMDM, we showed that TRIM33 deficiency altered pathways that regulate the late phases of BMDM activation and of *in vivo* LPS-mediated inflammatory response. Resolution of inflammation in M1 macrophages depends on two main categories of regulators [25]. The first group contains inhibitors of signaling pathways activated by microorganisms such as suppressors of cytokine signaling or A20. The second group contains transcriptional repressors such as ATF3 or peroxysome proliferator-activated receptors that repress the transcription of genes previously activated by microorganisms. Our results indicate that TRIM33 belongs to this second group of regulators. We have previously shown that TRIM33 expression was necessary for repression of the *Ifnb1* gene during late phases of BMDM activation. Here, we extend this result and show that TRIM33 targets genes at the late stages of LPS activation of BMDM. These genes are not part of a single pathway but are involved in lipid metabolism (*Abcg1*, *CD36* [26]), macrophage metabolism (*estrogen receptor α* (*Esr1*) [27]) and inflammatory response (purinergic receptor family, *ATP-gated P2X_7_ receptor* [28], -*P2y12 receptor* [29] and -*P2y14 receptor* [30], VEGF-A and the *OX40 ligand* (*Tnfsf4*) [31]).

In conclusion, our study reveals new important functions of TRIM33 in macrophage production and activation by LPS and links the TRIM33/PU.1 association to transcriptional changes that occur during the end of macrophage response to bacterial infection. Together with the role of TRIM33 during viral infection [15], these results pinpoint TRIM33 as a new clinical target for infectious diseases.

## EXPERIMENTAL PROCEDURES

### Mice and treatment

MxCre/*Trim33^−/−^* mice were previously described [12]. To generate *Trim33^−/−^*mice, *Trim33*^fl/fl^ C57BL/6-CD45.2 mice were crossed with Lysozyme-Cre C57/Bl6-CD45.2 mice (strain name: B6.129P2-Lyz2^tm1(cre)Ifo^/J, The Jackson Laboratory). Mice (6-16 weeks old) were injected intra-peritoneally with LPS (Sigma) according to mass per body weight (0.25mg/20g). Animal experiments were approved by the Committee on the Ethics of Animal Experiments according to the French Ministry of Agriculture (Act No.87-848, 19^th^ October 1987) and directive 2010/63/EU of the European parliament.

### Cell culture

Mouse bone marrow (BM) was flushed out of the tibia, femur and humerus using a syringe with PBS, filtered through a 70-μm mesh filter (BD Biosciences) to remove debris and pelleted by centrifugation, and cleared of adherent cells by incubation for 3 hours on tissue culture Petri dishes. BM cells were then cultured on tissue culture Petri dishes in IMDM supplemented with 10% FCS (Invitrogen), 1% penicillin streptomycin (PS) (Gibco), 10mM thioglycerol (Sigma) and 25ng/ml mouse CSF1 (Miltenyi Biotech) or 50 ng/ml GM-CSF (Miltenyi Biotech) or 20 ng/ml G-CSF (Miltenyi Biotec) for 7 days. On day 7, BMDM generated with CSF1 were activated with 100ng/ml LPS (Sigma) in IMDM supplemented with 2.5% FCS and 1% PS.

Resident peritoneal cells present without or 4 days after injection of thioglycollate (Sigma) were harvested with PBS and cultured for 2 hours in RPMI medium, supplemented with 10% FCS and 1% PS. All adherent cells were positive for F4-80 antigen (data not shown) and were studied as peritoneal macrophages. The next day, peritoneal macrophages were activated with 100ng/ml LPS (Sigma) in RPMI supplemented with 2% FCS and 1% PS.

For cell lines, myeloid 32D cells were cultured in RPMI adjusted to contain 10 mM HEPES and 1.0 mM sodium pyruvate, supplemented with 10% FCS, 1% PS and mouse Interleukin-3 (Becton Dickinson). RAW 264.7 cells were grown in DMEM supplemented with 10% FCS and 1% PS. All these cell lines were purchased in ATCC.

### Flow cytometry

Single cell suspensions of BM were depleted of red blood cells using an ammonium chloride solution (STEMCELL^TM^). Cells were incubated for 20 min with, PECy7 anti-B220, FITC anti-(F4-80, I-A(b), CD11c), PE anti-CD115, APC anti-Ly6C for monocytes; PECy7 anti-CD11b, FITC anti-(F4-80, I-A(b), CD11c), PE anti-Ly6G (eBioscience) for neutrophils. For progenitors populations, BM suspension cells were incubated with biotinylated lineage cell detection cocktail (Miltenyi Biotec) and revealed by streptavidin-eFluor450 (eBioscience) to discriminate between the positive and the negative lineages. The negative fraction was then labeled with FITC anti-CD34 (BD Biosciences), APC anti-c-Kit (BD Biosciences), PE anti-Sca-1 (BD Biosciences) and PECy7 anti-CD16/32 (eBioscience) antibodies for CMP and GMP analyses. For MDP and MOP analyses BM cells were labeled with APC-efluor780 anti-c-Kit, PE anti-CD115 and APC anti-CD135 antibodies (eBioscience). Peritoneal macrophages were labeled with PE anti-B220, FITC anti-F4-80, PECy7 anti-CD11b and APC anti CD11c antibodies (eBioscience). BMDM and dendritic cells from BM cultures were labeled with FITC anti-CD11c, PC7 anti-Ly6G, APC anti-CD11b, APC-Cy7 anti-F4-80 and PerCP5.5 anti-CD64.

Plasma levels of cytokines were quantified by flow cytometry (CBA Mouse inflammation kit, BD Biosciences).

Data acquisition was performed on FACS LSR II (BD Biosciences). Data were analyzed with FlowJo software version 9.5. Sorting cells were performed on FACS Aria II (BD Biosciences).

### Quantitative RT-PCR

Total RNA was extracted with the RNeasy Micro Plus kit (QIAGEN) and reverse transcribed with random primers and Superscript III (Invitrogen). Real-Time quantitative PCR was performed using the SYBR green in the 7900HT Fast Real-Time PCR System (Applied Biosystems). HPRT mRNA levels were used as an internal reference. Primer sequences are available upon request.

### cDNA microarrays and analysis

Total RNA was extracted with the RNeasy Micro kit (QIAGEN). Gene expression profiling was performed in biological duplicates on Mouse GE 8×60K Microarrays (Agilent) according to the manufacturer's instructions. Accession number (E-MTAB-1441, European Bioinformatic Institute (http://www.ebi.ac.uk/arrayexpress/).

The Bioconductor package LIMMA was used to assess statistical significance of differences in mRNA levels between *Trim33^−/−^* and Control macrophages. Transcripts with at least two-fold change and p-value<0.05 were retained as differentially regulated. Subsequent analyses were limited to probe sets matching a known transcript.

### Chromatin immunoprecipitation and sequencing

ChIP experiments were carried out with an anti-TRIM33 antibody (A301-059A, Bethyl) as previously described [12]. For sequencing, 10ng of purified DNA from ChIP was adapter-ligated, PCR amplified and sequenced by Illumina Genome Analyzer IIx according to the manufacturer's instructions (Illumina). Accession number (GSE43654).

The libraries were sequenced on the Illumina Genome Analyzer IIx as single-end 50 base reads following Illumina's instructions. Image analysis and base calling were performed using the Illumina Pipeline and sequence reads mapped to reference genome mm9/NCBI37 using Bowtie v0.12.7. In order to keep just the reads mapping to a unique position in the genome, Bowtie was run with the option –m 1. The option –best –strata were also used to get the best mapping position with the minimum of mismatch. In order to define the TRIM33 enriched genomic regions, peak calling was performed using MACS [32] with default parameters and using the corresponding IgG as control samples. Peaks were then annotated according to genomic features of Ensembl v67 using Homer [18] (http://homer.salk.edu/homer/). For co-localization studies, the TRIM33 peak positions of 32D and RAW cells and the PU.1 peaks in BMDM were intersected using BEDTools, requiring at least 1bp overlap. MEME-ChIP [33] (http://meme-suite.org/tools/meme-chip) was used to perform *de novo* motif discovery in TRIM33 peaks. Sequences spanning from -100bp to +100bp relative to the 600 highest enriched ChIP-Seq peak centers were extracted and repeat masked. The MEME algorithm was then used to identify motifs with a maximum size of 15bp and known motifs were derived from JASPAR and uniprobe databases. RSAT Matrix-Scan (http://rsat.ulb.ac.be/) was used to analyze TRIM33 and TRIM33/PU.1 peaks for transcription factor binding sites. Transcription factor matrices were extracted from Homer database and the occurrence was analyzed using a threshold p-value < 1e-4. The enrichment of Gene Ontology terms was calculated using DAVID tools or GREAT.

Comparisons between 32D and RAW ChIP-seq datasets at selected regions were performed using seqMINER [34]. Genomic coordinates were retrieved from Ensembl v67 and used as reference in the density array method. The collected data were subjected to k-means clustering using linear normalization. Data were represented as heat maps or average plots.

## SUPPLEMENTARY MATERIALS FIGURES AND TABLES


