# Supplementary material for: Macrophage production and activation are dependent on TRIM33
Source: Oncotarget. 2016 Dec 10;8(3):5111–22. doi: 10.18632/oncotarget.13872 (PMC5354896; doi:10.18632/oncotarget.13872)
Supplement: Supplementary file 1 [file oncotarget-08-5111-s001.pdf]

Macrophage production and activation are dependent on TRIM33

SUPPLEMENTARY FIGURES AND TABLES

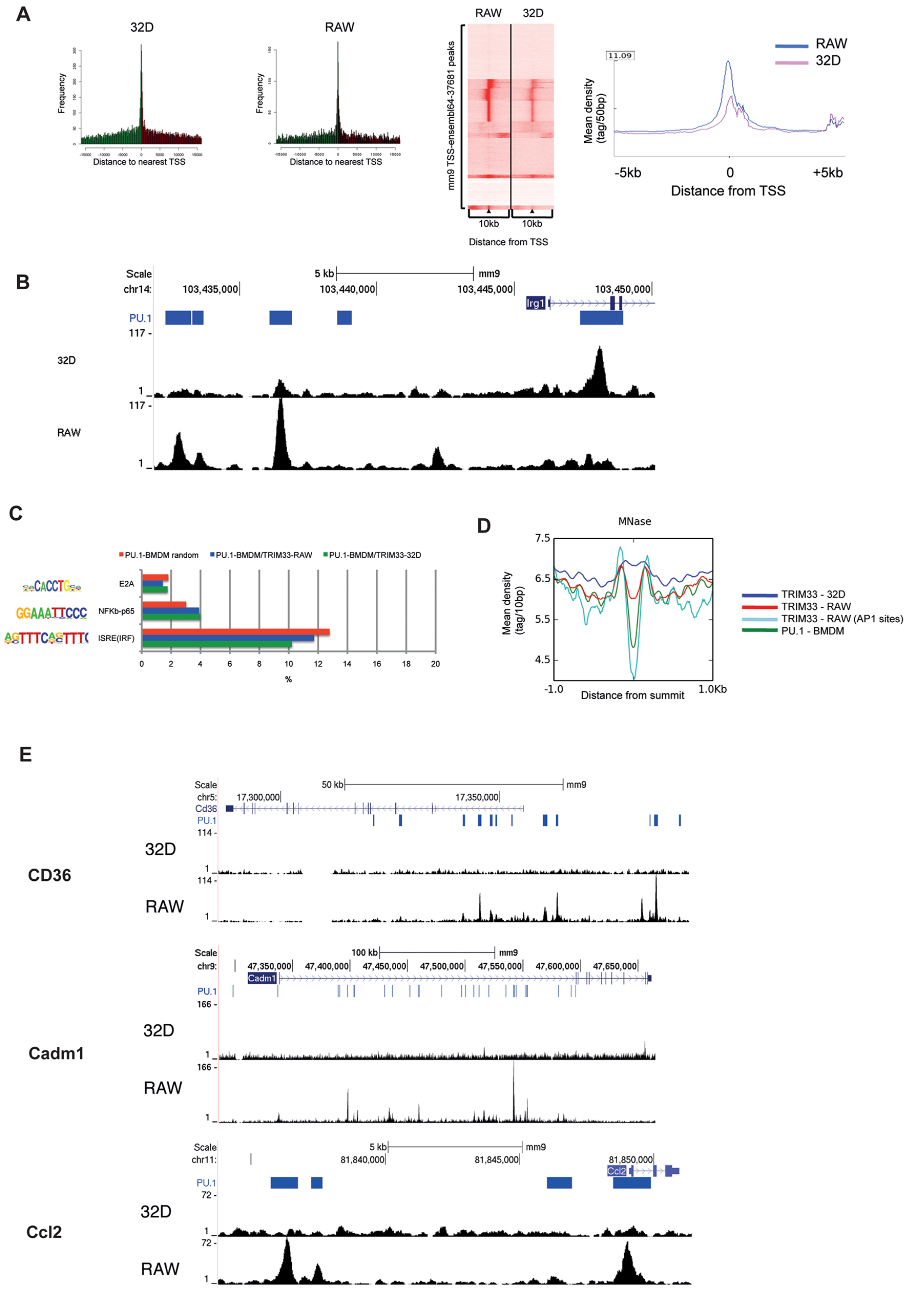

Supplementary Figure S1: A. (Left) Distribution of TRIM33 binding sites relative to nearest TSS in 32D and RAW cells. (Right) K-means clustering analysis and average profiles of TRIM33 ChIP-seq datasets at all mouse TSS ± 5kb, comparing 32D to RAW cells. **B.** UCSC genome browser image showing differential TRIM33 binding in 32D and RAW cells at the *Irg1* gene. **C.** Occurrence of the indicated transcription factor binding sites in the different TRIM33/PU.1 peak subsets in 32D (green) and RAW (blue) cells compared to their frequency in random PU.1-bound regions in BMDM (red) was analysed as in Figure 1D. **D.** MNase profiles in BMDM around the summit of TRIM33/PU.1 binding sites in 32D (dark blue) or RAW cells (red), around the summit of TRIM33/PU.1/AP1 binding sites in RAW cells (light blue) and around the summit of PU.1 peaks in BMDM (green). **E.** ChIP-seq analyses of TRIM33 in 32D and RAW cells for genes involved in inflammatory response.

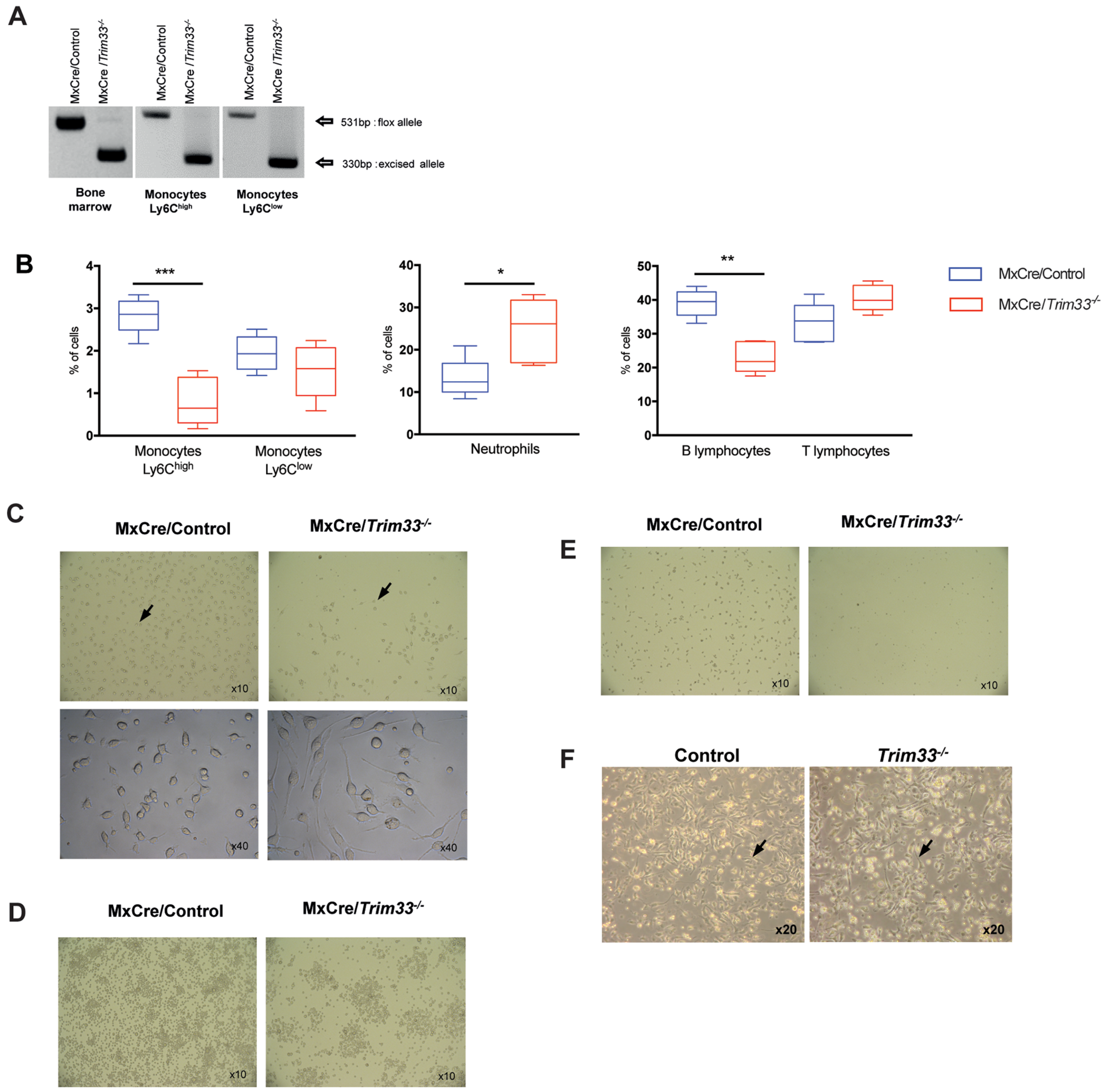

Supplementary Figure S2: A. Genomic PCR from MxCre/Control and MxCre/*Trim33*$^{-/-}$ bone marrow, monocytes Ly6C^{high} and monocytes Ly6C^{low} for *Trim33* floxed and *Trim33* excised allele. Only relevant bands are shown. **B.** Box plots of indicated blood cells of MxCre/Control and MxCre/*Trim33*$^{-/-}$ mice. **C.** Representative images of BMDM culture at different magnifications (10X upper panel and 40X lower panel) obtained from MxCre/Control and MxCre/*Trim33*$^{-/-}$ bone marrow treated with CSF-1. BMDM are pointed with arrow. **D.** Representative images of dendritic/macrophages cells obtained from MxCre/Control and MxCre/*Trim33*$^{-/-}$ bone marrow treated with GM-CSF. **E.** Representative images of culture obtained from MxCre/Control and MxCre/*Trim33*$^{-/-}$ bone marrow treated with G-CSF. **F.** Representative images of BMDM obtained from Control and *Trim33*$^{-/-}$ bone marrow. BMDM are pointed with arrow.

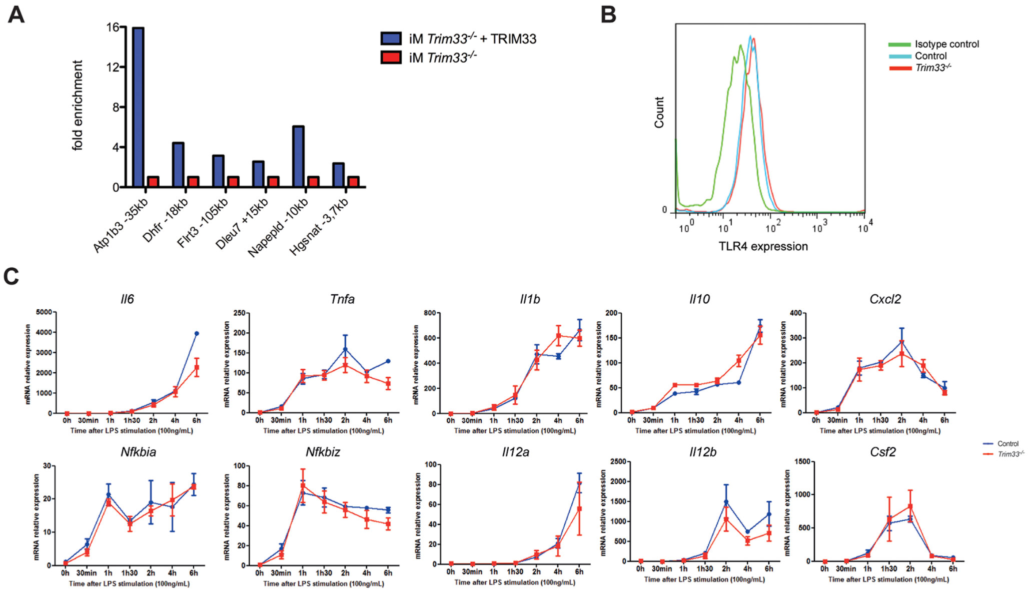

D

Go Term	Genes	pValue	*Trim33-/-* vs Control
GO: 0009611~response to wounding	SELP, PPARA, CXCL9, CCL8, F9, SGMS1, S1PR3, PROCR, MEFV, SERPINA1A, ID3, NOS2, KDM6B	8.4E-5	Down
GO: 0048285~organelle fission	SPC24, FIS1, SPC25, HAUS1, NUF2, BUB1, PEX11C, BIRC5, ANLN, ZWILCH, CDCA3, CENPH	2.3E-6	Up

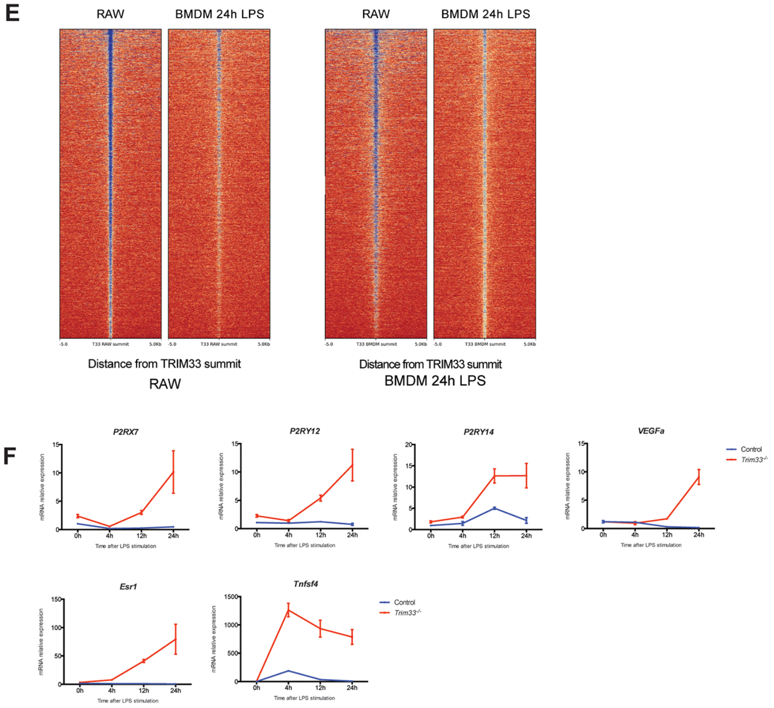

Supplementary Figure S3: A. ChIP-qPCR analyses of TRIM33 binding at indicated loci in immortalized *Trim33*$^{-/-}$ macrophages transduced with a lentivirus coding full length flag-TRIM33 (iM *Trim33*$^{-/-}$ + TRIM33). Data represent the fold enrichment over the TRIM33 signal obtained in immortalized *Trim33*$^{-/-}$ macrophages (iM *Trim33*$^{-/-}$). **B.** FACS analysis of TLR4 surface expression in Control (blue) and *Trim33*$^{-/-}$ (red) BMDM. **C.** Relative mRNA levels of several inflammatory genes at indicated time points after LPS activation (100ng/ml) of Control and *Trim33*$^{-/-}$ BMDM. Data are the average fold changes relative to untreated Control BMDM ± SEM. n=3. **D.** GO functional annotation analysis of the genes differentially regulated 4 hours after LPS activation of *Trim33*$^{-/-}$ BMDM. **E.** Heat maps showing binding of TRIM33 at the same regions in RAW cells and BMDM treated 24h with LPS. Data represent distribution of regions bound by TRIM33 in RAW cells and BMDM treated 24h with LPS, ± 5kb relative to TRIM33 summit peaks in RAW cells (left) and in BMDM treated 24h with LPS (right). **F.** Relative mRNA levels of the indicated genes during LPS activation (100ng/ml) of Control and *Trim33*$^{-/-}$ BMDM. Data are the average fold changes relative to untreated Control BMDM ± SEM, n=3.

A

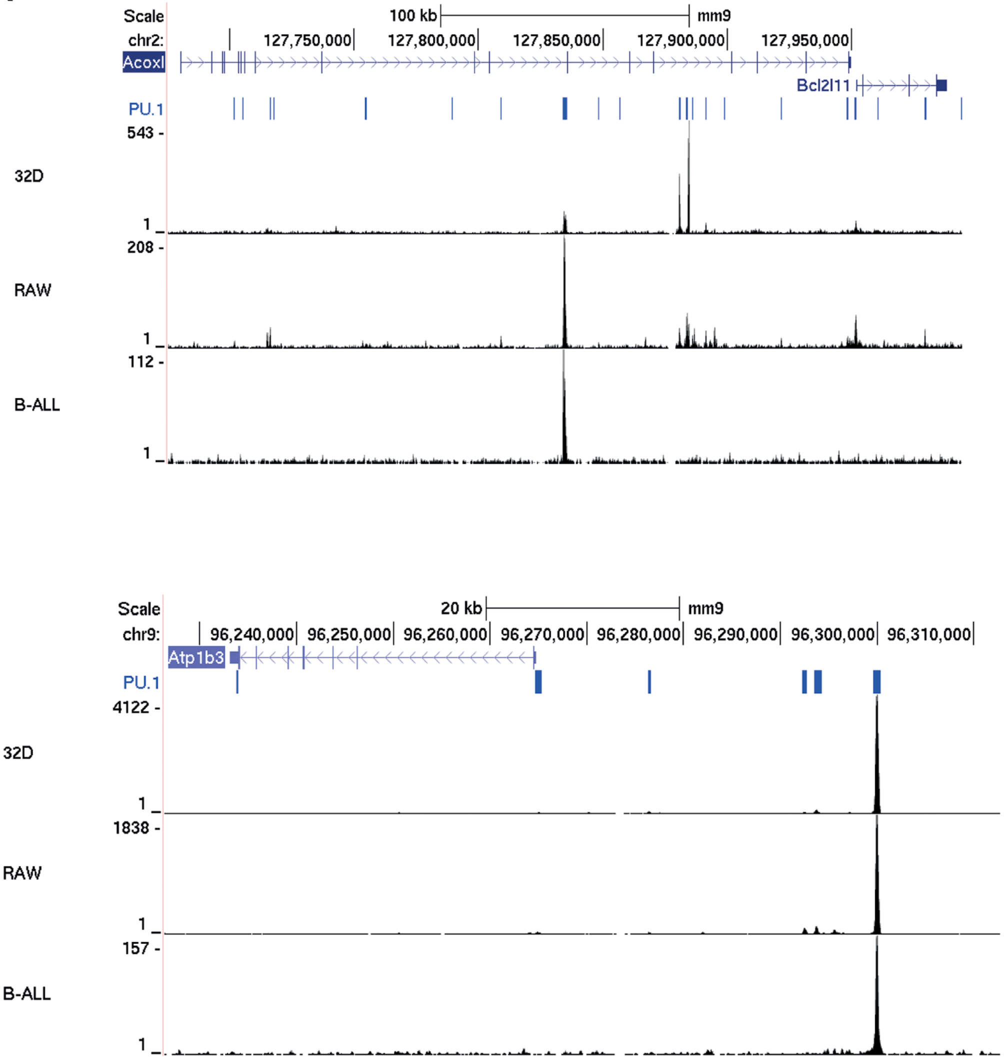

B

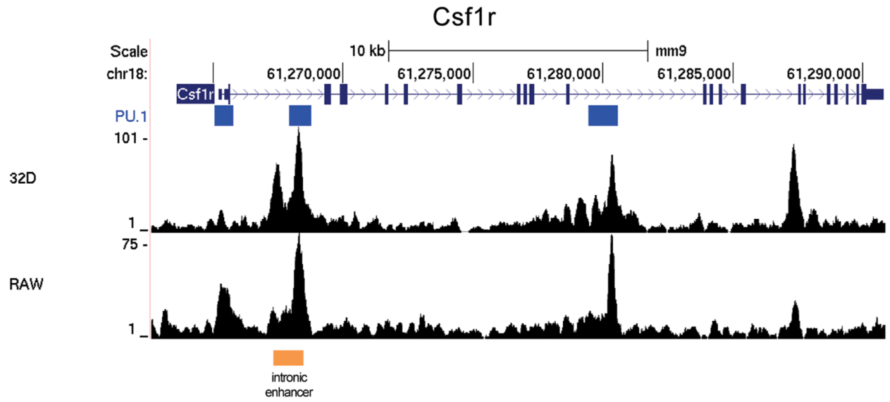

Supplementary Figure S4: A. ChIP-seq analyses of TRIM33 in 32D, RAW and B-ALL cells for *Acoxl*, *Bcl2l11/Bim* and *Atp1b3* genes. **B.** UCSC genome browser image showing TRIM33 binding in 32D and RAW cells at the *Csf1-R* gene. The position of the *Csf1-R* intronic enhancer is indicated in orange.

Table S1: Under- and over-expressed genes (FC 2) in untreated *Trim33*$^{-/-}$ BMDM. Genes with TRIM33 peaks in RAW cells are shown in bold

Down-regulated genes (Trim33$^{-/-}$ vs Control)			Up-regulated genes (Trim33$^{-/-}$ vs Control)		
Adam19	***Gpha2***	*Pfkl*	*Acot3*	***Heatr7b1***	***Rnaseh2b***
Ahrr	***Gpr68***	***Pkd1l2***	***Acoxl***	***Hgsnat***	***Rnf7***
Ak4	*Gsta3*	*Pla2g7*	*Apcdd1*	***Inpp1***	*Rpa2*
Antxr1	***Gstm2***	***Prkce***	*Armcx2*	***Ism1***	*Scml2*
Apoc1	***Gzmk***	***Prss48***	*Astn2*	*Itpr3*	***Sel1l3***
Apol7c	***H2-DMa***	***Rabgap1l***	***Atp1b3***	***Kcnab1***	***Slc17a5***
Bmf	*Hemt1*	*Rad1*	***Bcl2l15***	*Kctd19*	*Slc18a1*
Cap2	*Hist2h2be*	***Saa1***	*Btg4*	*Krt17*	***Slc26a4***
Cbr2	*Hunk*	***Saa3***	*Butr1*	***Lilra6***	*Slc6a16*
Ccdc48	***Ifi27l1***	***Serpina3f***	***Cadm1***	***Lpl***	*Slc9a10*
Ccl8	***Il1b***	*Serpinb3c*	***Carhsp1***	***Lrrc14b***	*Slfn4*
Cd3g	*Il28b*	*Serpinf1*	***Cblb***	***Mapk1ip1l***	*Snap25*
Celsr2	***Ildr2***	***Slc16a3***	***Ccdc41***	*Mapk4*	***St3gal5***
Cep63	***Itga9***	***Stam2***	*Cdc6*	***Msh3***	*Stfa1*
Cfh	*Lrrc2*	*Sult1a1*	***Cdk1***	***Napepld***	*Stfa2*
Cfhr2	***Mpp4***	***Tfrc***	*Cgn*	***Neo1***	***Stfa2l1***
Cib2	***Nnt***	***Thy1***	***Clec2d***	*Olfr1221*	***Stfa3***
Cxcl5	*Nova1*	***Tmem184b***	***Cmpk2***	*Olfr700*	*Tacr2*
Cxcl9	*Olfr103*	***Tmem37***	***Cttn***	*Olfr96*	***Tgfbi***
Cxcr6	*Olfr1316*	***Trib1***	***Def8***	***Oprm1***	***Tgm2***
Dbp	*Olfr285*	***Trim33***	*Defb36*	***Pgbd5***	***Tmtc2***
Emr4	*Olfr319*	*Tspo2*	***Dhfr***	*Pira11*	*Trav3-4*
Erdr1	*Olfr549*	*Vcam1*	***Dleu7***	*Pira2*	***Trim59***
Fcrls	*Olfr638*	***Vwf***	***Dlgap2***	*Pira6*	***Tubb3***
Fxyd2	*Olfr74*	***Zfp367***	***Dner***	*Pira7*	***Tuft1***
Fzd1	***Oplah***	***Zfp652***	***Efcab6***	***Plagl1***	***Ube2l6***
Gjc2	***Papss2***		***Epas1***	***Pou3f1***	***Usp54***
Gm8221	*Pdlim1*		*Fgf23*	*Prc1*	***Ust***
			Flrt3	***Prune2***	***Wdfy1***
			Fmr1nb	***Psd***	***Wdr92***
			Grip1	***Racgap1***	*Yipf7*

Table S2: Most under- or over-expressed genes (FC 4) in untreated *Trim33*$^{-/-}$ BMDM. Genes with TRIM33 peaks in RAW cells are shown in bold

Down-regulated genes (Trim33$^{-/-}$ vs Control)		Up-regulated genes (Trim33$^{-/-}$ vs Control)	
gene symbol	*mRNA*	*gene symbol*	*mRNA*
Saa3	*-6,47*	*Stfa1*	*175,58*
Cxcl9	*-4,91*	***Stfa3***	*123,56*
Fzd1	*-4,47*	***Flrt3***	*116,07*
		Dleu7	*114,75*
		Stfa2	*105,93*
		Stfa2l1	*78,24*
		Cgn	*58,29*
		Hgsnat	*29,63*
		Dhfr	*13,5*
		Lrrc14b	*7,9*
		Apcdd1	*7,81*
		Napepld	*7,77*
		Slfn4	*7,51*
		Acoxl	*7,26*
		Atp1b3	*5,61*
		Sel1l3	*5,2*
		Tubb3	*4,97*
		Neo1	*4,77*
		Tmtc2	*4,47*
		Pou3f1	*4,35*
		Cadm1	*4,33*
		Def8	*4,1*
		Rnaseh2b	*4*

Table S3: Under- or over-expressed (FC 2) genes in *Trim33*$^{-/-}$ BMDM after LPS activation. Genes with TRIM33 peaks in RAW cells are shown in bold

Down-regulated genes

0h, 24h	0h, 4h, 24h	4h, 24h		24h only	
Antxr1	*Cd3g*	*Abpd*	*Olfr207*	***Ascl2***	***Oaf***
Cxcl5	*Celsr2*	*Adam30*	*Olfr513*	*Bmp1*	*Olfr441*
Hist2h2be	*Cep63*	*Arl9*	*Olfr97*	*Btn1a1*	*Olfr559*
Itga9	*Emr4*	***Atp1a3***	***Osm***	***C3***	*Pogk*
Olfr638	*Erdr1*	***Atp2c2***	*Pde5a*	*Catsper3*	***Ppm1n***
Vcam1	*Gzmk*	***Bsn***	*Plagl1*	*Cd27*	*Ppp2r2c*
	Hemt1	*Cd2*	*Ppbp*	*Cd34*	***Prkcb***
	Ildr2	*Cd3d*	***Scml4***	*Cd96*	***Pstpip2***
	Lrrc2	*Cd74*	*Sdpr*	***Chchd7***	*Raver2*
	Nova1	*Cetn1*	*Selp*	*Chsy1*	*Rgs3*
	Olfr319	*Clec4n*	*Serpina1a*	*Ctdsp2*	***Rims3***
	Pdlim1	*Clu*	*Skap1*	***Cx3cr1***	*Scn1b*
	Pla2g7	*Cpne5*	*Spesp1*	***Dhcr24***	***Sema4a***
	Prss48	*Csf2*	*Sprn*	***Dot1l***	*Slc13a3*
	Rad1	*Cyp4a10*	***Stard4***	***Dusp16***	*Slc39a4*
	Serpinb3c	*Dub2a*	*Tcfcp2*	***Egr1***	***Smad6***
	Thy1	***Eif2c2***	*Tepp*	*F2r*	***Sqle***
	Trim33	***Emb***	***Tesk1***	*F5*	***Susd3***
	Tspo2	*Ephb2*	*Tnnc2*	*Fasl*	*Syt13*
	Zfp367	***F9***	***Treml1***	*Gbp8*	*Tbx21*
		Fam59b	*Upp1*	*Gimap3*	*Tgm3*
		Fxyd1	*Vmn1r217*	*Gimap8*	*Tmem145*
		Ggnbp1	*Vmn1r48*	*Gja1*	***Tmem176b***
		Gpc4	*Vmn1r52*	***Gng12***	*Trpc6*
		Gpihbp1	*Vmn1r87*	*Gp1ba*	***Uck2***
		Gpr6	***Vprbp***	***Gpr141***	*Xcl1*
		Grk4	*Zap70*	*Gsc2*	
		H2-Aa	*Zim3*	*H2-Eb1*	
		Hap1		*Hp*	
		Harbi1		***Idi1***	
		Hbb-bh1		*Kcnj5*	
		Ifitm1		***Klf3***	
		Ifng		*Klra22*	
		Igj		*Klra23*	

(*Continued*)

Down-regulated genes

0h, 24h	0h, 4h, 24h	4h, 24h	24h only
		Klra16	*Klra7*
		Lce1k	*Klrb1b*
		Mab21l3	*Lancl3*
		Mdh1b	*Lck*
		Ms4a4a	***Ldlr***
		Myh8	***Lox***
		Myo1b	*Lum*
		Necab2	*Ly6f*
		Nrg1	***Ly6i***

Over-expressed genes

0h, 24h	0h, 4h, 24h	4h, 24h	24h only			
Astn2	***Acoxl***	*Afp*	*Abat*	***Cytip***	*Kif9*	***Ptplad2***
Efcab6	*Apcdd1*	*Ajap1*	*Abca8b*	*Dao*	*Klkb1*	***Rab19***
Grip1	*Armcx2*	*Arhgap8*	***Abcg1***	*Dclre1a*	*Krt2*	*Ralyl*
Heatr7b1	***Atp1b3***	*Atf7ip2*	*Acss2*	*Dcxr*	*Krtap10-4*	***Rcan1***
Inpp1	***Cadm1***	*B3gnt3*	*Agt*	*Ddx4*	*Krtap16-8*	*Reck*
Kctd19	*Cgn*	***Camk2a***	***Ahnak***	***Diras2***	*Ksr2*	***Rgs1***
Neo1	***Dhfr***	*Cbr3*	***Aig1***	*Dnahc2*	*Lepr*	***Rhov***
Olfr1221	***Dleu7***	*Copz2*	***Ak8***	***Dnajb4***	*Lhb*	*Rnase6*
Olfr96	***Dlgap2***	***Cpe***	*Akap12*	Dpep2	*Lipn*	*Rnf144b*
Rnaseh2b	*Fgf23*	*Efr3b*	*Amac1*	*Egfl7*	***Lrrc27***	*Sat2*
Scml2	***Flrt3***	***Epb4.1l4b***	***Angptl3***	***Elovl2***	*Lrrc36*	*Sdk2*
Slc17a5	***Hgsnat***	***Esr1***	*Ankrd24*	*Erbb2*	***March10***	***Serpine1***
Slc6a16	*Ism1*	***Fam198b***	***Arl4a***	*Etnk2*	*Mex3b*	***Setd4***
Tgm2	*Kcnab1*	***Fis1***	***Arl4d***	*F8*	***Mgst3***	*Sgcb*
Tuft1	***Lilra6***	*Gabrr1*	***Arl5c***	*Fam13c*	*Mill2*	***Sgk1***
Usp54	*Lrrc14b*	*Galnt12*	***Armc4***	*Fam195a*	*Mllt3*	*Siah3*
Wdfy1	***Msh3***	***Hist2h2bb***	*Armcx6*	*Fam3b*	***Mmp12***	*Slc13a2*
	Napepld	*Idua*	***Art3***	*Fbn2*	*Mog*	*Slc16a12*
	Olfr700	***Lrrc51***	*Atp6v0d2*	*Fbp1*	***Msrb2***	*Slc46a1*
	Rnf7	***Nhedc2***	*Aym1*	***Fbxl20***	*Naip1*	***Spp1***
	Sell13	*Nipa1*	*B3gnt8*	*Fbxo39*	*Nfatc4*	*Ssxb9*
	Slc18a1	*Npdc1*	***Bank1***	***Fgd4***	*Nkain1*	***Stambpl1***
	Snap25	*Olfr738*	*Barx2*	*Fgf6*	***Nkiras1***	Stard5

(*Continued*)

Over-expressed genes

0h, 24h	0h, 4h, 24h	4h, 24h	24h only			
	Stfa1	***P2rx7***	***Bcl2l11***	*Fgfbp3*	*Nlgn2*	*Sucnr1*
	Stfa2	***P2ry12***	*Bgn*	*Folr2*	*Nos1*	*Syngr1*
	Stfa2l1	***Pacrg***	***Blvra***	*Ggt5*	*Olfr1211*	*Tas1r1*
	Stfa3	*Pde8b*	*Cacna1a*	*Ghr*	*Olfr122*	*Tcstv3*
	Tacr2	*Pla2g5*	*Cad*	***Glrp1***	*Olfr458*	*Tet1*
		Pygm	*Calcb*	*Gnaz*	*Olfr482*	*Tex9*
		Qpct	*Caly*	*Gng11*	*Olfr522*	***Timeless***
		Raet1c	*Ccdc46*	*Gpr37l1*	*Olfr713*	*Tmem17*
		Slc25a37	*Ccdc67*	*Gpx1*	*Olfr975*	***Tmem40***
		Snx7	*Ccl24*	*Gstk1*	*Ostn*	***Tmem86a***
		Speg	***Ccnf***	***Gsto1***	***P2ry13***	***Tmod1***
		Stard6	*Cd200r3*	***Gtpbp3***	*Pcbd1*	***Tnfsf4***
		Stk36	*Cd209d*	*Gyltl1b*	*Pitx3*	*Tppp3*
		Sycn	***Cd300lf***	*H2-M2*	*Plcxd2*	***Tpx2***
		Tgfb3	***Cd36***	*Hist1h4h*	*Plekha4*	***Trem2***
		Tmem29	***Cd72***	*Hmga2-ps1*	***Plk2***	***Trmt61b***
		Trio	*Cdkl5*	*Hspg2*	*Pnp2*	*Upb1*
		Trpc2	***Chrm3***	***Ifnb1***	***Ppp1r15a***	*Vangl2*
		Uprt	*Chst11*	***Il33***	***Prdx1***	***Vegfa***
		Wars2	*Cmbl*	*Irx4*	*Prelid2*	*Vmn2r4*
		Wnt6	*Cox6a2*	*Jph3*	*Prss29*	***Vrk2***
		Zfp286	*Cxcl14*	*Kcnh3*	***Ptgis***	*Wdfy2*
		Zfp644	*Cyp17a1*	***Kidins220***	*Ptgr1*	*Wscd2*
		Zranb3	*Cyp2c38*	***Kif5c***	*Ptk7*	
